# Supplementary material for: AFM for Nanomechanical Assessment of Polymer Overcoatings on Nanoparticle-Decorated Biomaterials
Source: Nanomaterials (Basel). 2024 Sep 11;14(18):1475. doi: 10.3390/nano14181475 (PMC11434162; doi:10.3390/nano14181475)
Supplement: Supplementary file 1 [file nanomaterials-14-01475-s001.zip › nanomaterials-3169711-supplementary.pdf]

# Supporting Information

## AFM for Nanomechanical Assessment of Polymer Overcoatings on Nanoparticle Decorated Biomaterials

**Jonathan Wood [1], Dennis Palms [2], Ruvini Dabare [1], Krasimir Vasilev [2,*] and Richard Bright [2,*]**

[1] Future Industries Institute, University of South Australia, Mawson Lakes, Adelaide, SA 5095, Australia; jcwood@swin.edu.au (J.W.); ruvini.dabare@unisa.edu.au (R.D.)
[2] College of Medicine and Public Health, Flinders University, Bedford Park, SA 5042, Australia; dennis.palms@flinders.edu.au
* Correspondence: krasimir.vasilev@flinders.edu.au (K.V.); richard.bright@flinders.edu.au (R.B.)

**Table S1.** Data were acquired using an NT-MDT NSG03 cantilever. Each overcoating measurement was performed using different cantilevers from the same batch as supplied by the manufacturers. Values for each cantilever calibration and setup are displayed in the supplementary table below. Free-air amplitude values at a magnitude of $10^{-15}$ are below the instrument's expected noise floor. However, the values presented are from instrumentation software.

| **Overcoating Thickness** | **0 Nm Overcoating** | **5 Nm Overcoating** | **10 Nm Overcoating** | **15 Nm Overcoating** | **20 Nm Overcoating** |
|---|---|---|---|---|---|
| Sensitivity (nm/V) | 29 | 30.5 | 33 | 24.8 | 25.4 |
| Q-factor | 158 | 168 | 162 | 161 | 157.6 |
| Free air Amplitude (m/√Hz) | $7.8 \times 10^{-15}$ | $9.4 \times 10^{-15}$ | $9.2 \times 10^{-15}$ | $8.6 \times 10^{-15}$ | $8.8 \times 10^{-15}$ |
| Spring constant (N/m) | 2.8 | 1.8 | 2 | 2.35 | 2.3 |
| Set Point (nm) | 27 | 26 | 29 | 22 | 22 |

**Base film variations**

A 20 nm-thick plasma-processed MePPOx base film functionalised with 68 nm Au NPs was selected as an antibacterial nanostructured surface due to the reasonable control of plasma time for adjusting polymer film thickness and for the ease of altering the ratio of NP size to the thickness of the overcoating film. Other plasma-derived polymers and Au NP sizes were analysed for their overall roughness in both tapping and contact mode topography over a 5 x 5 μm scan area. A force curve test was performed to compare adhesion and friction forces using a SiN conical cantilever tip. All analysis was performed with four NT-MDT NSG30 cantilevers with a typical Sader spring constant in the 30-40 N/m range and a torsional spring constant in the 6E-9 N/m range, as shown in Table S2(a) below. Au NP of three sizes, 16, 32, and 68 nm diameter, was placed on three plasma polymer coatings, with and without a 20-nanometre-thick MePPOx overcoating. These polymers are shown in Table S2(b), with variations in the NP size, whether they have a ~20 nm overcoating of the same base film material, and the cantilever used, as presented in Table S1 (a).

**Table S2**. (A) NTMDT NSG30 cantilever calibration values. (B) The range of samples tested and compared. Sample numbers are labelled by the polymer base film, MePPOx (M), Allylamine (AA), Acrylic Acid (AC), the Au NP diameter of either 16, 32, or 68 nm, and if the sample has a 20nm overcoating of the

same polymer as the base film, with no overcoating (N), and with an overcoating (Y). Four NT-MDT NSG30 cantilevers were used, labelled C1–C4.

**A**

| Cantilever Number | Resonant Frequency (kHz) | Normal Sader SC (N/m) | Torsional SC (E-9 N/m) | Q-factor |
|---|---|---|---|---|
| C1 | 281.7 | 32 | 6.45 | 512 |
| C2 | 281.6 | 32.8 | 6.6 | 531 |
| C3 | 273.9 | 30.2 | 6.1 | 507 |
| C4 | 281 | 32.4 | 6.6 | 531 |

**B**

| Sample Number | Base Material | Overcoating (Y/N) | Cantilever Number |
|---|---|---|---|
| M16N | MePPOx | N | C1, C2 |
| M16Y | MePPOx | Y | C1, C2 |
| M38N | MePPOx | N | C1, C2 |
| M38Y | MePPOx | Y | C1, C2 |
| M68N | MePPOx | N | C1, C2 |
| M68Y | MePPOx | Y | C1, C2 |
| AA16N | Allylamine | N | C3, C4 |
| AA38N | Allylamine | N | C3, C4 |
| AA68N | Allylamine | N | C3, C4 |
| AC16N | Acrylic Acid | N | C3, C4 |
| AC38N | Acrylic Acid | N | C3, C4 |

Roughness values were compared not only between tapping and contact topography modes but also for samples with and without plasma overcoating applied at a thickness of 20 ± 5 nm, Table S2–S7. Most of the sample's topography was scanned using AFM, and each contained a small amount of dirt, dust, plasma polymer remnants, etc. that adhered to the substrate at some point during processing. Data processing software has tools that allow for the removal of these anomalies that change roughness values. The *-marked sample values highlight the roughness values of the surface filtered by Gwyddion software to remove surface anomalies. A significant contrast in roughness values occured between the two topography modes, with and without overcoating, and in the software-filtered values. This was expected owing to differences in tip–substrate topography forces, the change in Au NP interaction with the tip with and without an applied overcoating and removing topographical features through software processing.

**Table S3.** (A) Roughness values of MePPOx with **16 nm** Au NP measured and compared in tapping and contact topography mode, and (B) Force curve values of the force load (the force of the cantilever tip's contact with the substrate), the adhesion force of the cantilever tip as it retracts from the surface, and the friction force as measured through LFM data. The average friction force for the M16Y* sample shown in (b) was not able to be calculated due to computer failure.

**A**

| Roughness value | Sample M16N | | Sample M16Y | | Sample M16Y* | |
|---|---|---|---|---|---|---|
| | Tapping | Contact | Tapping | Contact | Tapping | Contact |
| **RMS (nm)** | 3.1 | 2.7 | 24.3 | 2 | 5.8 | 1.3 |
| **Ra (nm)** | 2.2 | 2.3 | 10.8 | 1.5 | 4.7 | 1 |
| **Peak height (nm)** | 96 | 18 | 518 | 53 | 59 | 7 |

**B**

| Force Curve Values | M16Y | M16Y* |
|---|---|---|
| **Force load (nN)** | 0.44 | 2.116 |
| **Adhesion force (nN)** | 0.37 | 1.536 |
| **Av friction force (nN)** | 0.33 | - |

**Table S4**. (A) Roughness values of MePPOx with 32 nm Au NP measured and compared in tapping and contact topography modes, and (B) force curve values of the force load, the adhesion force of the cantilever tip as it retracts from the surface, and friction force as measured through LFM data.

**A**

| | Sample M32N | | Sample M32N* | | Sample M32Y | | Sample M32Y* | |
|---|---|---|---|---|---|---|---|---|
| **Roughness value** | Tapping | Contact | Tapping | Contact | Tapping | Contact | Tapping | Contact |
| **RMS (nm)** | 31.3 | 6.8 | 16.8 | 3.3 | 28.3 | 5.3 | 3 | 5.3 |
| **Ra (nm)** | 19.4 | 5 | 12 | 2.8 | 23.5 | 4.2 | 15.5 | 2.6 |
| **Peak height (nm)** | 559 | 73 | 366 | 27 | 322 | 64 | 238 | 14 |

**B**

| Force curve values | M32N | M32Y |
|---|---|---|
| **Force load (nN)** | 0.18 | 0.33 |
| **Adhesion force (nN)** | 0.27 | 0.37 |
| **Av friction force (nN)** | 0.24 | 0.22 |

**Table S5.** (A) Roughness values of MePPOx with **68 nm** Au NP measured and compared in tapping and contact topography modes, and (B) force curve values of the force load, the adhesion force of the cantilever tip as it retracts from the surface, and friction force as measured through LFM data.

**A**

| | Sample M68N | | Sample M68N* | | Sample M68Y | | Sample M68Y* | |
|---|---|---|---|---|---|---|---|---|
| **Roughness value** | Tapping | Contact | Tapping | Contact | Tapping | Contact | Tapping | Contact |
| **RMS (nm)** | 63.3 | 2 | 12.7 | 0.73 | 27.5 | 3.1 | 12.3 | 1 |
| **Ra (nm)** | 26.2 | 1.3 | 9.1 | 0.56 | 16.3 | 2.1 | 8.6 | 0.8 |
| **Peak height (nm)** | 1006 | 70 | 147 | 7 | 522 | 72 | 256 | 6 |

**B**

| Force Curve Values | M68N | M68Y |
| --- | --- | --- |
| **Force load (nN)** | 0.65 | 0.59 |
| **Adhesion force (nN)** | 0.58 | 0.68 |
| **Av friction force (nN)** | 0.5 | 0.62 |

**Table S6**. Roughness values of MePPOx without AuNP measured and compared in tapping and contact topography modes. Additionally, force curve values of the force load, the adhesion force of the cantilever tip as it retracts from the surface, and friction force as measured through LFM data are shown.

| Roughness value | Tapping | Contact | Force curve values | |
| --- | --- | --- | --- | --- |
| **RMS (nm)** | 4.78 | 4.33 | **Force load (nN)** | 0.96 |
| **Ra (nm)** | 3.4 | 3.51 | **Adhesion force (nN)** | 0.48 |
| **Peak height (nm)** | 153 | 85 | | |

**Table S7**. Roughness values of Allylamine with (A) 16 nm Au NP, (B) 32 nm Au NP, (C) 68 nm Au NP measured and compared in tapping and contact topography modes, and (D) force curve values of the force load, the adhesion force of the cantilever tip as it retracts from the surface, and friction force as measured through LFM data.

**A**

| | Sample AA16N | | Sample AA16N* | |
| --- | --- | --- | --- | --- |
| **Roughness value** | **Tapping** | **Contact** | **Tapping** | **Contact** |
| **RMS (nm)** | 15.3 | 3.5 | 8.7 | 1.3 |
| **Ra (nm)** | 12.9 | 2 | 7.3 | 1.1 |
| **Peak height (nm)** | 92 | 76 | 58 | 8 |

**B**

| | Sample AA32N | | Sample AA32N* | |
| --- | --- | --- | --- | --- |
| **Roughness value** | **Tapping** | **Contact** | **Tapping** | **Contact** |
| **RMS (nm)** | 6.8 | 2.3 | 2.9 | 1.4 |
| **Ra (nm)** | 2.5 | 1.7 | 1.8 | 1.2 |
| **Peak height (nm)** | 339 | 48 | 97 | 8 |

**C**

| | Sample AA68N | | Sample AA68N* | |
|---|---|---|---|---|
| **Roughness value** | **Tapping** | **Contact** | **Tapping** | **Contact** |
| **RMS (nm)** | 31 | 2 | 27.3 | 1 |
| **Ra (nm)** | 25.3 | 1.3 | 22.7 | 0.7 |
| **Peak height (nm)** | 292 | 52 | 225 | 8 |

**D**

| Force curve values | AA16N | AA32N | AA68N |
|---|---|---|---|
| **Force load (nN)** | 0.16 | 0.2 | 0.13 |
| **Adhesion force (nN)** | 0.2 | 0.27 | 0.07 |
| **Av friction force (nN)** | 0.28 | 0.1 | 0.5 |

**Table S8**. (A) Roughness values of Acrylic Acid with 16 and 32 nm Au NP measured and compared in tapping and contact topography mode, and (B) force curve values of the force load, the adhesion force of the cantilever tip as it retracts from the surface, and friction force as measured through LFM data.

**A**

| | Sample AC16N | | Sample AC16N* | | Sample AC3NY | | Sample AC32N* | |
|---|---|---|---|---|---|---|---|---|
| **Roughness value** | **Tapping** | **Contact** | **Tapping** | **Contact** | **Tapping** | **Contact** | **Tapping** | **Contact** |
| **RMS (nm)** | 5.7 | 2.1 | 5.7 | 1.3 | 18 | 23 | 17.6 | 11.2 |
| **Ra (nm)** | 4 | 1.58 | 4 | 1 | 14.7 | 16 | 14.7 | 9.5 |
| **Peak height (nm)** | 198 | 45 | 41 | 8 | 255 | 203 | 132 | 76 |

**B**

| Force curve values | AC16N | AC32N |
|---|---|---|
| **Force load (nN)** | 0.13 | 0.12 |
| **Adhesion force (nN)** | 0.4 | 0.37 |
| **Av friction force (nN)** | 0.32 | 0.4 |
